# Prognostic Value of Serum Exosomal AHCY Expression in Hepatitis B-Induced Liver Cirrhosis

**DOI:** 10.3389/fmed.2021.777452

**Published:** 2021-11-08

**Authors:** Ling Tong, Cuilin Yan, Minjie Wang, Jiajia Yang, Hongmei Wang, Ying Wang

**Affiliations:** ^1^Department of Clinical Laboratory, The First Affiliated Hospital, College of Medicine, Zhejiang University School of Medicine, Hangzhou, China; ^2^Department of Infection Management, The Affiliated Suzhou Hospital of Nanjing Medical University, Suzhou Municipal Hospital, Gusu School, Nanjing Medical University, Suzhou, China

**Keywords:** adenosylhomocysteinase (AHCY), exosomes, hepatitis B-related cirrhosis (HBV-LC), prognosis predictor, chronic hepatitis B (CHB)

## Abstract

**Objective:** We aimed to investigate serum exosomal adenosylhomocysteinase (AHCY) expression in hepatitis B-induced liver cirrhosis (HBV-LC) patients and to determine the prognostic value of serum exosomal AHCY.

**Methods:** We collected serum samples from 100 patients with chronic hepatitis B (CHB) and from 114 HBV-LC patients to test serum exosomal AHCY expression using ELISA.

**Results:** Compared with the CHB and Grade A and B HBV-LC groups, the level of exosomal AHCY expression was significantly higher in the HBV-LC group [376.62 (291.50–448.02) vs. 248.12 (189.28–324.63), *P* > 0.001] and the Grade C HBV-LC group [408.70 (365.63–465.76) vs. 279.76 (215.16–336.07), *P* > 0.001], respectively. Serum exosomal AHCY expression and MELD score had a significant positive correlation (*r* = 0.844, *P* < 0.001). Survival curve analysis showed that patients with low exosomal AHCY expression had significantly longer survival than patients with high exosomal AHCY expression (*P* = 0.0038). The receiver operating characteristics (ROC) curve showed that the area under the curve (AUC) value for the mortality prediction ability of serum exosomal AHCY in HBV-LC patients was 0.921, which was higher than the values for the MELD score (AUC 0.815) and Child-Pugh classification (AUC 0.832), with a sensitivity and specificity of 93.41 and 76.00%, respectively.

**Conclusions:** The serum exosomal AHCY level is a novel potential prognostic biomarker in HBV-LC patients, which may be great significance for the prognosis of HBV-LC patients.

## Introduction

Hepatitis B virus (HBV) is a pathogen that causes a generalized epidemic that constitutes a global problem (1). Worldwide, approximately 2 billion people have been infected by HBV, nearly 400 million people carry the HBV, and almost 20 million individuals have chronic hepatitis B (CHB). In hepatitis B-induced liver cirrhosis (HBV-LC) following chronic HBV infection, hepatocytes gradually become necrotic; moreover, this necrosis leads to the fibronodular proliferation of hepatocytes and hepatic tissues, and the normal liver lobules are replaced by pseudobullets (2, 3). In cirrhosis, the recurrent and continuous progression of hepatic fibrosis and inflammation can lead to hepatic dysfunction, ascites, esophagogastric varices and variceal bleeding, portal hypertension, acute kidney injury, and hepatic encephalopathy; with disease progression, cirrhotic patients can develop life-threatening hepatocellular carcinoma (HCC) (4, 5). In 2015 in China, there were 460,000 and 420,000 primary HCC cases and HCC-related deaths, respectively, which constituted more than 50% of the total global incidence and mortality of HCC; thus, HCC has become a public health problem that endangers human health (6, 7). The early diagnosis and treatment of cirrhosis are of great importance for the prognosis of patients with HCC. At present, imaging, histopathological examination, and serum index assessment are routinely used as the main diagnostic modalities to assess the stage of cirrhosis (8).

Originally identified from the supernatant of cultured sheep reticulocytes, exosomes are vesicles (diameter 30–150 nm; density 1.10–1.18 g/ml) (9) that contain various proteins, mRNA, and miRNA lipids; thus, exosomes can be used as carriers for information transfer and modulate various *in vivo* biological activities and thereby provide a novel route for cell communication (10, 11). Exosomes facilitate the study of the pathophysiology, diagnosis, treatment, and prognosis of many diseases because of their unique lipid bilayer membrane structure that protects their biological properties and helps to maintain their stability at extremely low temperatures and for long durations (12–14). The liver is one of the most important organs in the human body, and many liver cells can either secrete exosomes or are exosome-target cells, such as hepatocytes, bile duct epithelial cells, hepatic stellate cells (HSC), mononuclear macrophages, natural killer T lymphocytes, lymphocytes, etc. (15, 16). Furthermore, exosomes secreted by different cells have different functions. Exosomes are involved in the pathogenesis of HCC, viral hepatitis, liver fibrosis, and alcoholic and non-alcoholic fatty liver disease, and, increasingly, exosomal proteins and miRNAs have been identified as potential biomarkers of various diseases (17, 18).

S-adenosyl-L-homocysteine hydrolase (SAHase), a highly conserved enzyme, catalyzes the reversible hydrolysis of S-adenosyl-L-homocysteine (SAH) to homocysteine (Hcy) and adenosine (Ado) (19) and thereby regulates the intracellular adenosylhomocysteinase (AHCY) concentration, which is considered important for transmethylation reactions. In a mouse model of liver injury for screening a novel serum marker, Vazquez et al. found that the AHCY concentration significantly increased with the increasing severity of liver injury (20).

This study was performed to determine the expression of the prognostic assessment value of serum exosomal AHCY in HBV-LC patients.

## Materials and Methods

### Patients

We retrospectively collected serum samples from 100 CHB and 114 HBV-LC patients who were admitted to the First Hospital of Zhejiang University School of Medicine, the Second People's Hospital of Yancheng City, and the Fifth People's Hospital of Wuxi from August 2019 to August 2020, with a follow-up duration of 3 months.

The CHB case was defined as: there is a history of hepatitis B or HBsAg positive for more than 6 months, and HBsAg and/or HBV DNA are still positive. The diagnostic criteria for cirrhosis was as follows:

#### Diagnostic Basis of Compensated Cirrhosis (One Out of Four Required)

(1) Histologically consistent with the diagnosis of cirrhosis. (2) Endoscopy demonstrates esophagogastric varices or ectopic varices of digestive tract, except for non-cirrhotic portal hypertension. (3) Imaging examinations such as B-super, LSM, or CT indicate the characteristics of cirrhosis or portal hypertension. For example, splenomegas and portal veins ≥1.3 cm, LSM assays meet the diagnostic boundaries of cirrhosis for different etiology. (4) For those without histology, endoscopy, or imaging examination, the following inspection indicators indicate the presence of cirrhosis (two out of four required).

(1) PLT < 100×109/L, and there is no other reason to explain; (2) Serum albumin < 35g/L, excluding other causes such as malnutrition or kidney disease; (3) INR > 1.3 or PT extension (deactivation of thrombosis or anticoagulants for over 7 days); (4) AST/PLT Ratio Index (APRI): adult APRI score >2. Attention should be paid to the effects of factors such as antisense drugs on APRI.

#### Diagnostic Basis of Decompensated Cirrhosis

On the basis of cirrhosis, complications of portal hypertension and/or impaired liver function occur. (1) Have the diagnostic basis of cirrhosis; (2) Portal hypertension related complications occur, such as ascites, esophageal varicose vein rupture bleeding, sepsis, hepatoencephalopathy, liver and kidney syndrome, and so on.

#### Re-compensated Cirrhosis and Reversal of Cirrhosis

Clinical studies have shown that patients with decompensated HBV and HCV-related cirrhosis can significantly improve liver function through effective antiviral treatment, including improving liver compensatory function, reducing portal hypertension related complications, and ultimately avoiding liver transplantation, similar to compensated cirrhosis. Liver function re-compensation during antiviral therapy is more common in patients with HBV-associated cirrhosis than in patients with HCV-associated cirrhosis. At present, the definition of re-compensation for decompensated cirrhosis is still unclear and controversial. In short, patients with decompensated cirrhosis, due to effective control of etiology, effective treatment of complications or prevention, will no longer appear decompensated cirrhosis events (ascites, gastrointestinal bleeding, hepatic encephalopathy) in a longer period of time (at least 1 year), but still exist compensated cirrhosis clinical and laboratory characteristics, which is considered “re-compensated cirrhosis.”

We excluded patients with: (1) non-HBV-related cirrhosis; (2) a history of relevant drug treatment within the last month; and (3) severe heart, brain, kidney disease, thrombocytopenia, and so on.

The Child-Pugh grading system comprises five items (21)—ascites, albumin, total bilirubin, hepatic encephalopathy, and prothrombin time—that constitute a total score in the range of 5–15 points, based on which it is categorized as grades A, B, and C (total score: 5–8, 9–11, and 12–15 points, respectively).

The MELD score includes three objective indices (22): serum bilirubin concentration, serum creatinine concentration, and INR, as well as the etiology of liver cirrhosis. The MELD score is calculated as follows:

MELD score = 9.57 × In (serum creatinine) + 3.78 × In (serum bilirubin) + l1.2 × In (INR) + 6.43 × (The etiology of liver cirrhosis, wherein alcoholic and cholestatic etiologies were assigned 0 points, and the rest were scored 1 point).

The study protocol was approved by the Ethics Committee of the First Hospital of Zhejiang University School of Medicine (No. 2017003), and informed consent from each patient was obtained.

### Serum Exosome Separation

Before the serum exosome separation, we washed the qEV separation column (qEV original 35 and 70 nm, Izon Christchurch, New Zealand) with at least 10 ml PBS (1×), which we then extracted from the top of the sieve plate with a pipette before removing the bottom sliding cap from the column. Then, we added 500 μl sample to the top of the sieve plate and immediately replaced the bottom sliding cap, and collecting 0.5 ml of the fraction. When the final sample is placed inside the top sieve plate of the column (at the same level), add 2.5 ml PBS (1×) to obtain a final void volume of 3 ml. The 1.5 ml liquid, which is the higher purity exosome solution, is retained after the void volume is collected in a 1.5-ml EP tube.

### Transmission Electron Microscopy

For transmission electron microscopy (TEM), we diluted the exosome and filtered the 5 μl-sample by adding it drop by drop onto the copper net and then incubated it for 5 min at room temperature. Thereafter, we used blotting paper on one side to absorb the excess liquid, added a drop of 2% uranium peroxide acetate to the copper net, and re-incubated the net for 1 min at room temperature. Following this step, we used blotting paper on one side of the net to absorb the excess liquid, allowed the net to dry for approximately 20 min at room temperature, and then observed and photographed the shape of the exosome under the electron microscope.

### Nanoparticle Tracking Analysis

For the nanoparticle tracking analysis (NTA), the frozen samples were thawed in a water bath at 25°C and placed on ice. The exosomes were diluted with PBS (1×) and used directly for the NTA (ZetaVIEW S/N 17-310) assay. The NTA software (ZetaView 8.04.02) was used to analyze the movement of particles and to calculate the number of exosomes.

### Western Blotting

For the gel preparation, we used a 1.5-mm glass plate with a 15-well sample comb to prepare a 12% isolate gel and a 5% concentrate gel according to the molecular weight of the target protein. Electrophoresis was carried out at a steady pressure difference of 80 V until the loading buffer (indicator) entered the separation gel and then changed to a steady pressure of 120 V, which was maintained until the loading buffer reached the bottom of the gel to terminate electrophoresis. We selected a PVDF membrane that had a pore size of 0.22 μm and maintained a constant current of 200 mA for a transfer time of 90 min with 5% skimmed milk powder diluted in PBST, closed for 1 h. The PVDF membrane was washed three times, for 10 min each, using PBST and then placed in the hybridization cassette. The corresponding antibody was added, and the membrane was placed on a decolorization shaker overnight at 4°C. Then, the mix was shaken slowly to bring it to room temperature. The primary antibody [Annexin V (sc-393669, 1:1,000), CD9 (sc-13118, 1:2,000), Tsg101 (sc-7964, 1:2,000), and CD63 (sc-5275, 1:1,000); Santa Cruz Biotechnology, Inc., Texas, USA] was removed and the membrane was washed three times using PBST for 10 min each time; the secondary antibody was added into the hybridization cassette along with the membrane, which was then placed on a shaker and slowly shaken and incubated at room temperature for 1 h. Then, the secondary antibody was removed and the membrane was washed three times using PBST, for 10 min in each wash cycle. We added the appropriate amount of ECL luminescent solution and used program one in the digital imaging system to continuously photograph the membrane for a maximum duration of 1 min.

### ELISA

For the ELISA < the residual cells were removed from the plasma samples and the cell fragments were diluted with 1 × PBS (1:500 diluted) and the exosomes were precipitated with 100 ml RIPA lysis buffer on ice for 30 min. The samples were diluted with PBS (1:3 diluted) after shaking and mixing. The ELISA plate that was previously coated with the AHCY antibody was taken out, and we added blank control solution to one well and solutions of gradient concentration to seven wells. The diluted exosome samples constituted a 100-μl solution. After incubation at 37°C for 60 min, the liquid in the well is discarded, and the plate is rotated and dried. Then, we added 100 μl Solution A to the plate, covered it with the film, incubated the plate in an oven at 37°C for 1 h, and then washed the plate three times. We added 100 μl Solution B to the plate, covered the plate with film, baked the plate in the oven at 37°C for half an hour, and then washed the plate five times. Next, we added 100 μl TMB substrate solution and colored it in the dark at 37°C for 20 min, and 50 μl of the termination reaction solution was added to the plate. The absorbance value was detected using the microplate reader at 450 nm wavelength.

### Statistical Analysis

Data were statistically analyzed using SPSS 22.0, and the measurement data are expressed as (x ± s). One-way ANOVA and the SNK-q test for two-way comparison were used to determine the intergroup differences. Pearson linear correlation analysis and logistic one-way risk factor analysis were used to evaluate the predictive ability of plasma exosome-derived AHCY. We calculated the survival rate with the Kaplan Meier method. ROC curve analysis was used to assess the prognostic value of exosomal AHCY levels in HBV-LC patients. Differences were considered statistically significant at *P* < 0.05.

## Results

### Characteristics of Exosomes

**Figure 1:**
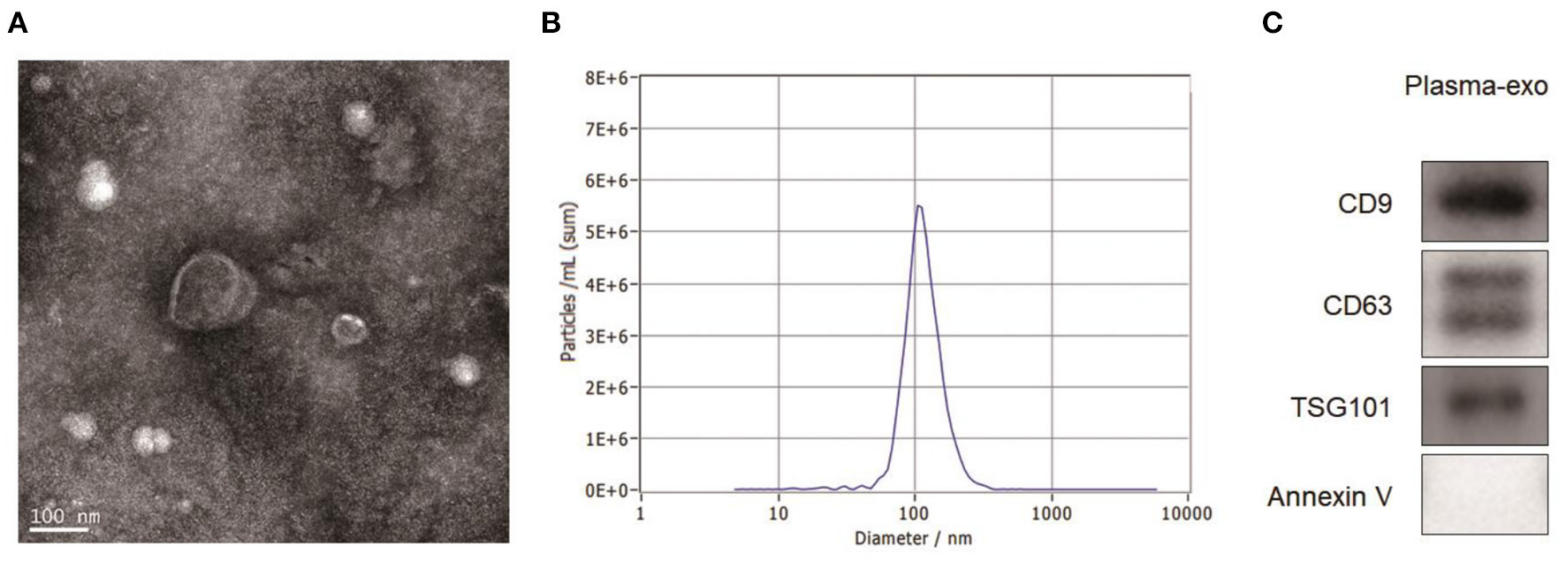
Exosome characterization. **(A)** TEM images showed that aggregates of exosomes were distributed while connected. The exosomes had a diameter of 100–200 nm and were shaped like a double disk-like vesicular structure that was completely covered with a lipid envelope. **(B)** The results of exosome particle size detected by NTA showed that the median value of the overall particle size was approximately 100 nm, and particle size was mainly distributed between 50 and 200 nm. **(C)** Western blotting showed the positive expression of the exosome marker proteins Annexin V, CD9, Tsg101, and CD63 in the exosome group.

The results of the TEM observation are shown in Figure 1A. Against a clear background, aggregates of exosomes were distributed while connected. The exosomes had a diameter of 100–200 nm and were shaped like a double disk-like vesicular structure that was completely covered with a lipid envelope. The results of exosome particle size detected by NTA are shown in Figure 1B. The median value of the overall particle size was approximately 100 nm, and particle size was mainly distributed between 50 and 200 nm. Western blotting showed the positive expression of the exosome marker proteins Annexin V, CD9, Tsg101, and CD63 in the exosome group (Figure 1C).

### Level of Exosomal AHCY Expression in HBV-LC Patients

**Figure 2:**
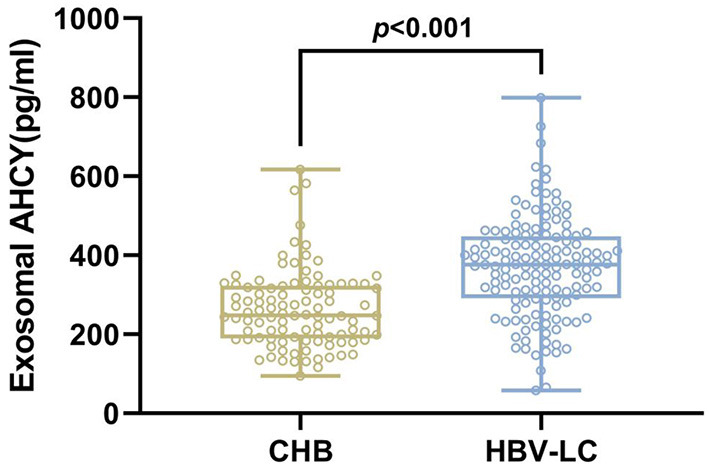
Expression level of exosomal AHCY in HBV-LC patients.

We evaluated the level of exosomal AHCY expression in HBV-LC patients (*n* = 141) and compared the expression levels with those in the CHB (*n* = 100) group. Table 1 shows the baseline characteristics of the CHB and HBV-LC groups. In the CHB group (age, mean ± SD: 53.43 ± 10.88 years), there were 61 male and 39 female participants; in the HBV-LC group (age, mean ± SD: 54.06 ± 11.99 years), there were 79 male and 62 female participants. The intergroup differences in age and sex were not statistically significant (*P* > 0.05). Compared with the CHB group, the level of exosomal AHCY expression was significantly higher in the HBV-LC group [376.62 (291.50–448.02) vs. 248.12 (189.28–324.63), *P* < 0.001; Figure 2].

**Table 1:** Baseline characteristics of CHB and HBV-LC groups.

**Variables**	**CHB**	**HBV-LC**	***P***
	**(*n* = 100)**	**(*n* = 141)**	
Age (years)	53.43 ± 10.88	54.06 ± 11.99	0.678
Gender (M/F)	61/39	79/62	0.441
WBC (10^9^/L)	4.2 (3.0–5.5)	7.0 (4.9–11.1)	<0.001
HB (g/L)	127 (112–141)	110 (89–126)	<0.001
PLT (×10^9^/L)	84 (52–132)	70 (42–114)	0.014
ALT (U/L)	31 (18–68)	79 (40–164)	<0.001
AST (U/L)	38 (26–87)	103 (56–177)	<0.001
TBIL (μmol/L)	20.6 (14.7–40.9)	178.0 (50.5–388.0)	<0.001
DBIL (μmol/L)	9.7 (6.3–19.3)	190.9 (53.8–299.5)	<0.001
ALB (g/L)	35.12 ± 7.09	30.00 ± 5.44	<0.001
BUN (mmol/L)	5.20 (4.28–7.66)	10.40 (5.56–18.16)	<0.001

### Correlation Between the Serum Exosomal AHCY Expression and the Child-Pugh Class and MELD Score in the HBV-LC Group

**Figure 3:**
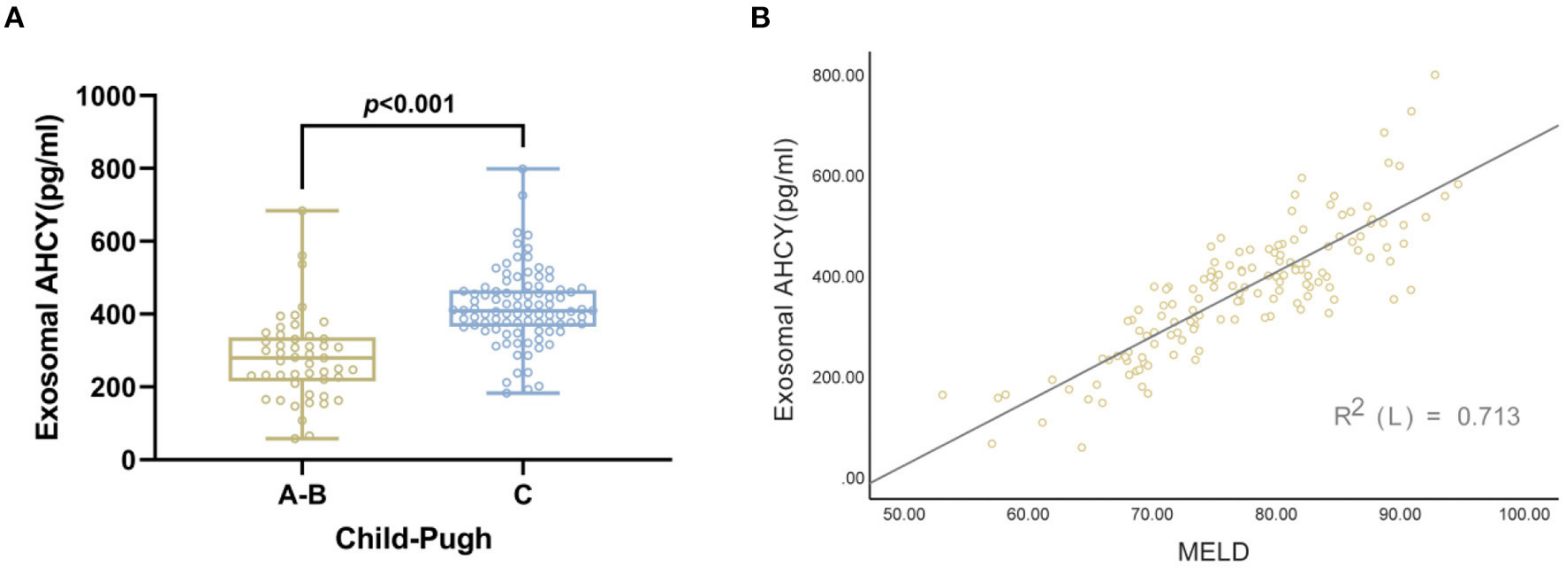
Correlation between serum exosomal AHCY level and child Pugh and MELD score in HBV-LC patients group. **(A)** The level of serum exosomal AHCY in HBV-LC patient with grade C group was significantly higher than that in HBV-LC patient with Grade A, B group [408.70 (365.63–465.76) vs. 279.76 (215.16–336.07), *P* > 0.001]; **(B)** there was a significant positive correlation between serum exosomal AHCY levels and MELD score (*r* = 0.844, *P* < 0.001).

Following the Child-Pugh classification, the HBV-LC patients were assigned to the Grade A, B, and C groups. Compared with the Grade A and B HBV-LC groups, the level of exosomal AHCY expression was significantly higher in the Grade C HBV-LC group [408.70 (365.63–465.76) vs. 279.76 (215.16–336.07), *P* < 0.001; Figure 3A]. In addition, we found a significant positive correlation between the serum exosomal AHCY level and the MELD score (*r* = 0.844, *P* < 0.001; Figure 3B).

### Correlation Between the Serum Exosomal AHCY Level and Clinical Parameters in the HBV-LC Group

**Figure 4:**
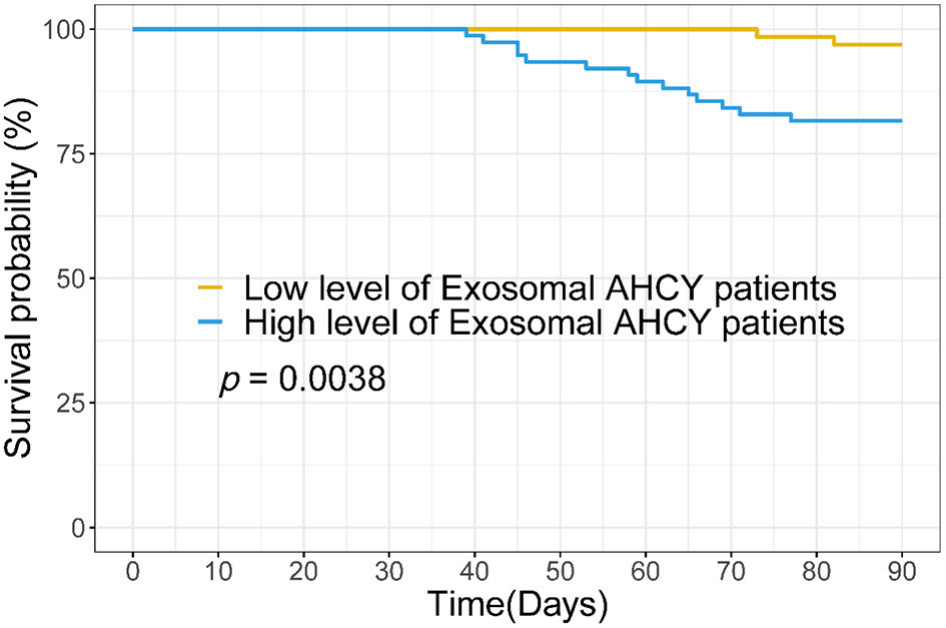
Survival curve analysis the relationship between serum exosomal AHCY level and survival time for HBV-LC patients.

Using the mean exosomal AHCY expression level (370.21) in the HBV-LC group as a cutoff point, we assigned the HBV-LC patients into the high expression and low expression groups based on exosomal AHCY expression. Table 2 shows the baseline characteristics of the two subgroups. The levels of WBC, HB, ALT, AST, TBIL, DBIL; the INR and PT; and the MELD score and Child-Pugh grade of the patients in the high expression group were significantly higher than those in patients in the low expression group; however, the PLT count was significantly lower in patients in the high expression than in the low expression group. The survival curve analysis showed a significantly longer survival time for patients in the low expression group than for patients in the high expression group (*P* = 0.0038; Figure 4).

**Table 2:** Baseline characteristics of exosomal AHCY high and low expression in HBV-LC group.

**Variables**	**Total**	**Exosomal AHCY high expression**	**Exosomal AHCY low expression**	***P***
	**(*n* = 141)**	**(*n* = 76)**	**(*n* = 65)**	
Age (years)	54.06 ± 11.99	53.29 ± 11.56	54.95 ± 12.50	0.413
Gender (M/F)	79/62	40/36	39/26	0.380
WBC (10^9^/L)	7.0 (4.9–11.1)	8.0 (5.7–11.4)	6.6 (3.6–10.3)	0.038
Hb (g/L)	110 (89–126)	116 (95–133)	107 (80–123)	0.044
PLT (×10^9^/L)	70 (42–114)	62 (35–104)	80 (53–120)	0.044
ALT (U/L)	79 (40–164)	103 (54–178)	62 (28–127)	0.004
AST (U/L)	103 (56–177)	118 (72–231)	72 (32–152)	<0.001
TBIL (μmol/L)	178.0 (50.5–388.0)	296.0 (146.8–472.8)	52.0 (19.0–201.5)	<0.001
DBIL (μmol/L)	190.9 (53.8–299.5)	253.5 (143.4–354.6)	55.0 (17.5–216.5)	<0.001
ALB (g/L)	30.00 ± 5.44	29.31 ± 5.38	30.81 ± 5.43	0.104
BUN (mmol/L)	10.40 (5.56–18.16)	11.82 (7.29–18.75)	8.60 (5.05–15.85)	0.072
Cr (μmol/L)	103 (72–182)	119 (76–194)	94 (70–128)	0.062
UA (μmol/L)	283 (174–468)	289 (182–491)	273 (164–446)	0.604
INR	1.84 (1.41–2.43)	2.18 (1.83–2.64)	1.46 (1.22–1.84)	<0.001
PT (s)	21.6 (16.8–27.8)	25.3 (21.5–31.8)	17.1 (14.5–21.1)	<0.001
BA	50 (28–77)	51 (28–80)	46 (28–74)	0.457
AFP (ng/ml)	69.5 (12.0–161.8)	73.0 (18.2–170.4)	65.6 (5.6–153.1)	0.375
PCT	1.29 (0.58–2.44)	1.16 (0.69–2.16)	1.57 (0.42–2.63)	0.791
ESR	14 (6–29)	13 (5–29)	17 (7–29)	0.618
CRP (mg/L)	21.5 (11.2–69.2)	21.6 (13.2–64.7)	19.3 (9.0–78.8)	0.513
MELD score	77.18 ± 8.33	82.23 ± 5.77	71.28 ± 6.88	<0.001
Child-Pugh	10 (9–11)	11 (10–12)	9 (7–10)	<0.001

### Prognosis Predictive Ability Based on the Serum Exosomal AHCY Level in HBV-LC Patients

**Figure 5:**
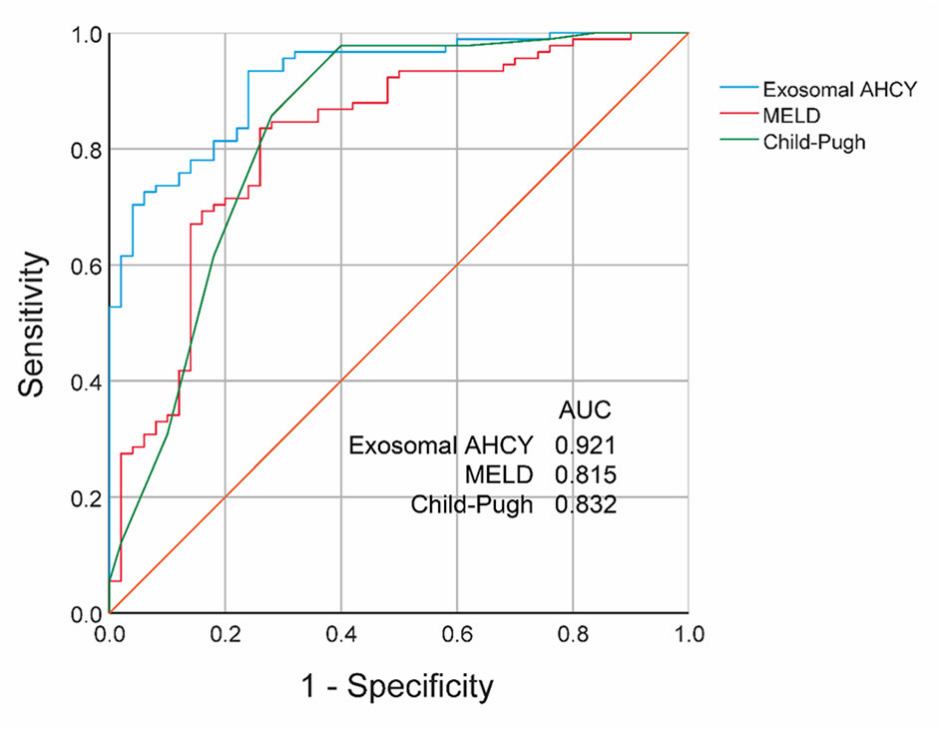
Prognosis predictive ability based on the serum exosomal AHCY level in HBV-LC patients.

Finally, we evaluated the prognosis prediction ability based on the serum exosomal AHCY expression in HBV-LC patients. The receiver operating characteristics (ROC) curve showed an area under the curve (AUC) value of 0.921 for serum exosomal AHCY expression in predicting the mortality risk of HBV-LC patients, which was higher than the AUC of the MELD score (0.815) and the Child-Pugh classification (0.832) (Figure 5). The sensitivity and specificity of serum exosomal AHCY expression in prognosis prediction were 93.41 and 76.00%, respectively.

## Discussion

Cirrhosis following an HBV infection is a chronic disease wherein the body, after HBV invasion, undergoes gradual liver cell necrosis that results in the fibronodular hyperplasia of liver tissue and the replacement of normal liver lobes by false lobes (23). In cirrhosis, repeated and continuously progressive liver fibrosis and inflammation induce loss of liver function, which results in decompensated liver function, ascites, esophageal gastric varices and variceal bleeding, portal hypertension, acute kidney injury, hepatic encephalopathy, and even HCC as well as several serious complications. In China, there were 460,000 primary liver cancer cases and 420,000 liver cancer-related deaths in 2015. The incidence of primary liver cancer in China in 2015 exceeded 50% of the total global incidence of primary liver cancer, which has emerged as a public health problem that endangers human health. Thus, the early diagnosis and treatment of cirrhosis are of great significance for the prognosis of patients.

In mammals, AHCY is the only enzyme that mediates the reversible catalysis of SAH to Ado and cysteine (24). The earliest manifestation of alcohol-related liver disease is steatosis, which is characterized by the accumulation of lipid droplets in liver cells. Arumugam et al. (25) demonstrated that many pathological changes, including steatosis, are associated with the alcohol-induced increase in SAH hepatocytes. A study that investigated the impaired Hcy metabolism in patients with alcohol-related liver disease from Taiwan (26) showed that impaired Hcy metabolism may disrupt antioxidant status. The role of alcohol-induced endoplasmic mesh stress in SREBP regulation and fatty liver, as well as the exact mechanism of beetroot protection, include decreased Hcy and SAH levels or increased S-adenosine methionine concentrations. Stender et al. (27) found that AHCY deficiency is associated with early-onset HCC. Vazquez et al. (20) screened novel serum markers in a mouse model of liver injury and demonstrated that AHCY expression significantly increased with the increase in the degree of liver injury. However, there are no studies of the role of exosomal AHCY in HBV-LC.

In this study, exosomes in the serum samples from patients with CHB and HBV-LC were separated, and TEM, NTA, and Western blotting were used to identify the characteristics of the exosomes. Transmission electron microscopy showed that exosomes had an interconnected aggregated distribution in a clear background, a diameter of 100–200 nm, and a complete lipid envelope, and were shaped as a double-disk vesicle. Nanoparticle tracking analysis showed an overall median exosome particle size of approximately 100 nm (range 50–200 nm). Western blotting revealed the positive expression of Annexin V, CD9, Tsg101, and CD63 in the exosome group. The exosomal AHCY levels of patients with HBV-LC were significantly higher than those of patients with CHB.

The Child-Pugh classification and the MELD score are important evaluation criteria in patients with severe liver disease. Therefore, we separately analyzed the correlation between the serum exosomal AHCY level and the abovementioned two assessment systems. The HBV-LC patients with Grade C cirrhosis had a significantly higher serum exosomal AHCY level than the HBV-LC patients with Grade A and B cirrhosis. In addition, we found a significant positive correlation between the serum exosomal AHCY level and the MELD score The above-described results indicated that the serum exosomal AHCY level has a good correlation with the two classic scoring models. Furthermore, we studied the correlation between the serum exosomal AHCY level and clinical parameters in the patients in the HBV-LC group and in the high expression and low expression subgroups that were based on exosomal AHCY expression. The levels of WBC, HB, ALT, AST, TBIL, and DBIL; the INR and PT; and the MELD score and Child-Pugh grade were significantly higher in patients with high expression of exosomal AHCY; however, the PLT count was significantly lower in patients with high exosomal AHCY expression than the PLT count in patients with low exosomal AHCY expression. Survival curve analysis showed that the survival time of patients with low exosomal AHCY expression was significantly longer. Finally, we evaluated the prognosis prediction ability of exosomal AHCY in HBV-LC patients. The ROC curve showed that the AUC value of serum exosomal AHCY for predicting the mortality risk of HBV-LC patients was higher than the AUC values of the MELD score and the Child-Pugh; moreover, exosomal AHCY expression showed good sensitivity and specificity in predicting the prognosis of HBV-LC patients.

This study has some limitations. First, the sample size of the study patients was not large; therefore, the predictive value of serum exosomal AHCY expression requires evaluation in multicenter, large studies. Second, this study lacked a control group for a controlled evaluation to verify whether the absence of exosomal AHCY expression is specific for the prognosis prediction in CHB or HBV-LC patients. Thirdly, our study did not involve the relevant mechanism research of AHCY involved in the development of disease.

In summary, our study showed that serum exosomal AHCY expression in HBV-LC patients is significantly upregulated and shows a good correlation with the Child-Pugh classification and the MELD score for prognosis prediction. HBV-LF patients with low exosomal AHCY expression had longer survival. The AUC value of serum exosomal AHCY in predicting the mortality risk of HBV-LC patients was higher than the AUCs of the MELD score and the Child-Pugh classification, which may facilitate the establishment of serum exosomal AHCY expression as a novel prognostic biomarker in HBV-LC.

## Data Availability Statement

The original contributions presented in the study are included in the article/Supplementary Material, further inquiries can be directed to the corresponding author/s.

## Ethics Statement

The studies involving human participants were reviewed and approved by the Ethics Committee of the First Hospital of Zhejiang University School of Medicine (No. 2017003). The patients/participants provided their written informed consent to participate in this study. Written informed consent was obtained from the individual(s) for the publication of any potentially identifiable images or data included in this article.

## Author Contributions

LT directed and supervised the study and revised the manuscript. CY and MW designed and performed most of the experiments. JY and HW participated in some experiments, analyzed the data, and completed the figures. LT and YW wrote the manuscript. All authors have read and approved the final manuscript.

## Conflict of Interest

The authors declare that the research was conducted in the absence of any commercial or financial relationships that could be construed as a potential conflict of interest.

## Publisher's Note

All claims expressed in this article are solely those of the authors and do not necessarily represent those of their affiliated organizations, or those of the publisher, the editors and the reviewers. Any product that may be evaluated in this article, or claim that may be made by its manufacturer, is not guaranteed or endorsed by the publisher.

## Abbreviations

HBV: hepatitis B virus
CHB: chronic hepatitis B
HBV-LC: hepatitis B-induced liver cirrhosis
HSC: hepatic stellate cells
AHCY: adenosylhomocysteinase
NTA: nanoparticle tracking analysis
TEM: transmission electron microscopy.
