# Supplementary figures and images for: Prognostic Value of Serum Exosomal AHCY Expression in Hepatitis B-Induced Liver Cirrhosis
Source: Front Med (Lausanne). 2021 Nov 8;8:777452. doi: 10.3389/fmed.2021.777452 (PMC8606640; doi:10.3389/fmed.2021.777452)

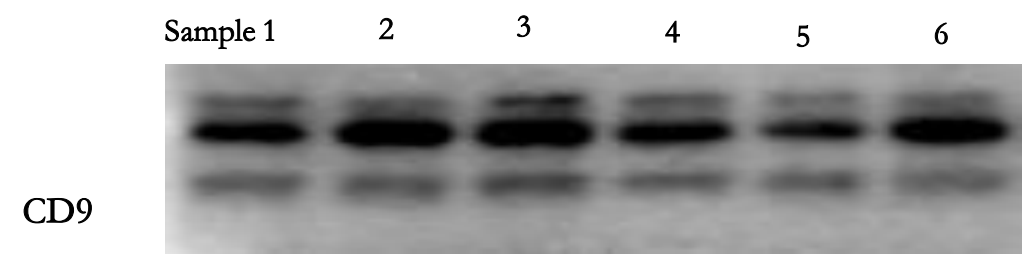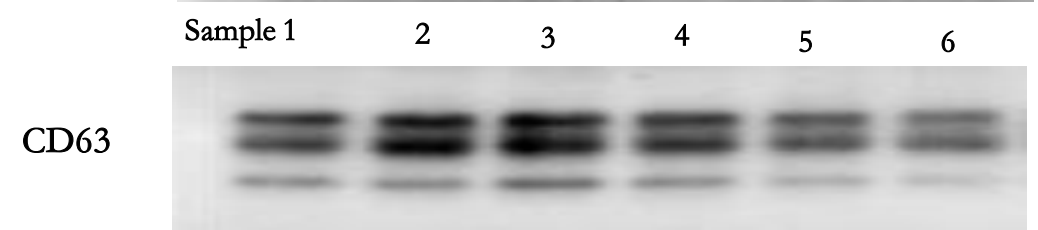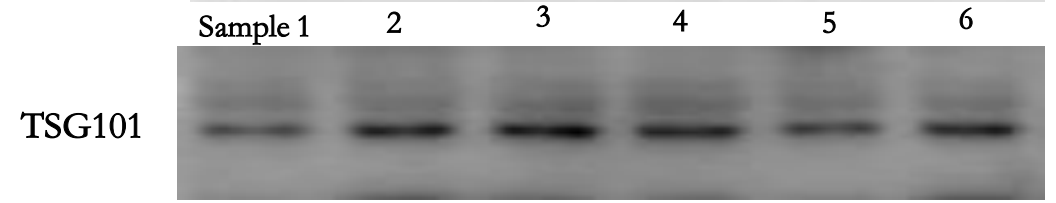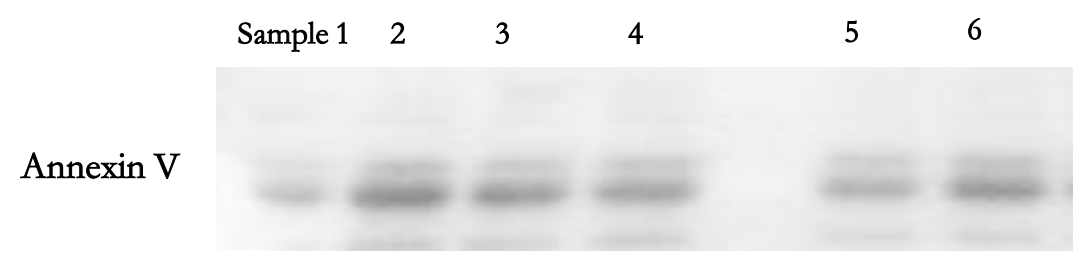

Supplement: Supplementary file 2 [file Data_Sheet_2.PDF]
